# Bridging Clinical Care and Self-Management: Impact of Nursing Module Based on WHO’s Universal Self-Care Framework in Women with Endometriosis

**DOI:** 10.3390/healthcare14050664

**Published:** 2026-03-06

**Authors:** Hajer I. Motakef, Niven Basyouni, Salam Bani Hani, Emran A. Abu Aqoulah, Bahia Galal Abd Elrazik Siam, Soha Kamel Mosbah Mahmoud, Layla Salem Alshammari, Elham Saeed Abdo Moqbel, Isis Emile Gohar, Zainh I. Motakef, Fatima Diab, Mokhtar A. Almoliky, Bushra Alshammari, Awatif Alrasheeday

**Affiliations:** 1 Department of Maternal and Child Health, College of Nursing, University of Hail, Hail 55473, Saudi Arabia; ha.matekef@uoh.edu.sa (H.I.M.);; 2 Nursing Department, Irbid National University, Irbid 22110, Jordan or n.basyouni@inu.edu.jo (N.B.);; 3 Department of Obstetrics & Gynecologic Nursing, Faculty of Nursing, Alexandria University, Alexandria 21527, Egypt; 4 Department of Medical Surgical Nursing, College of Nursing, University of Hail, Hail 55473, Saudi Arabia; b.siam@uoh.edu.sa (B.G.A.E.S.);; 5 Department of Community Health Nursing, College of Nursing, University of Hail, Hail 55473, Saudi Arabia; 6 Department of Nursing, Faculty of Medicine and Health Sciences, University of Hodeida, Al Hudaydah P.O. Box 3114, Yemen; 7 Department of Administration, College of Nursing, University of Hail, Hail 55473, Saudi Arabia

**Keywords:** endometriosis, self-care practices, nursing intervention, pain reduction, health maintenance, WHO guidelines, quasi-experimental study, women’s health

## Abstract

**Highlights:**

**What are the main findings?**

- At one month, the study group showed significantly higher self-care scores than the control group (69.2 ± 11.6 vs. 58.3 ± 8.83; *p* = 0.001).
- At three months, self-care practices further improved in the study group compared with the control group (76.4 ± 16.5 vs. 61.5 ± 12.2; *p* = 0.001).
- A strong negative correlation was observed between self-care and symptom severity at one month (r = −0.70, *p* < 0.001).
- This negative correlation was even stronger at three months (r = −0.83, *p* < 0.001), indicating that better self-care was associated with reduced symptoms.

**What are the implications of the main findings?**

- The findings will address inadequate self-care compliance and persistent pain among women with endometriosis, alongside the limited use of structured, nursing-led self-care interventions in routine care.
- The findings will impact gynecological and nursing practice settings, particularly benefiting women with endometriosis by enhancing self-management, pain control, and quality of life through nursing-led care models.

**Abstract:**

**Aims:** This study aims to assess the effect of a structured universal self-care practices module on improving self-care compliance and health maintenance behaviors among women with endometriosis, and to determine if it reduces pain severity. **Design:** A quasi-experimental design was used. **Methods:** A total of 90 women confirmed a diagnosis of endometriosis, who were free from any chronic medical or gynecological comorbidities, and who had not received any pain relief pharmacological interventions. **Results:** The study and control groups were comparable at baseline regarding socio-demographic and clinical characteristics (*p* > 0.05). Following the intervention, the study group demonstrated significant improvements in universal self-care practices compared to the control group at one month (M = 69.2 ± 11.6 vs. 58.3 ± 8.83; t = −4.93, *p* = 0.001) and three months (M = 76.4 ± 16.5 vs. 61.5 ± 12.2; t = −4.89, *p* = 0.001). A strong negative correlation was found between self-care and symptom severity at one month (r = −0.70, *p* < 0.001) and three months (r = −0.83, *p* < 0.001), indicating that improved self-care was associated with reduced symptoms. **Conclusions:** This study highlights the vital role of nursing-led, WHO-based self-care interventions in improving compliance and reducing pain among women with endometriosis. Integrating such programs into routine care can enhance self-management and overall quality of life. **Patient or Public Contribution:** Integrating individualized, nursing-led self-care programs into routine endometriosis management can improve symptom control through ongoing education and follow-up. Nurses play a pivotal role in empowering women’s self-management, while adopting the WHO Universal Self-Care Framework can strengthen gynecological care policies and practices.

## 1. Introduction

Endometriosis represents one of the prevalent gynecological conditions characterized by the ectopic presence of endometrial-like tissue beyond the uterus [1]. It is a chronic, estrogen-dependent inflammatory disorder that is often diagnosed among women who experience a range of debilitating symptoms, including pelvic pain, dysmenorrhea, dyspareunia, menorrhagia, dyspepsia, and infertility [2]. According to the World Health Organization statistics, a substantial proportion of women of reproductive age are impacted by this condition, although the prevalence rates may vary based on diagnostic practices, awareness, and geographic location [3].

The detrimental effects of endometriosis surpass merely physical symptoms, often leading to a compromised quality of life, emotional distress, lower daily activities, and sexual dissatisfaction [4]. Other concurrent conditions include fatigue, anxiety, depression, diminished work effectiveness, and broader psychosocial challenges [5]. As a recurring condition without a definitive cure, management strategies focus on symptom control, fertility preservation, and enhancement of quality of life [6]. Treatment methods generally consist of pharmacological therapies such as hormonal treatments and pain relievers, surgical procedures, and non-pharmacological approaches [7]. Self-care and self-management are increasingly recognized as vital in chronic disease management, enabling individuals to reduce symptoms and improve psychological and social well-being [8].

According to Cummins [9], women with endometriosis often use various self-care strategies, such as dietary changes, heat therapy, exercise or yoga, rest, stress-reduction methods, alternative therapies, and lifestyle adjustments.

Despite the widespread adoption of self-care behaviors, the evidence supporting their effectiveness remains inconsistent. Small sample sizes, variability in intervention types, potential biases, and a lack of standardized outcome measures limit several studies [10,11,12]. Furthermore, there is a notable scarcity of research on structured educational or nursing-based interventions specifically designed to support self-care in women with endometriosis [13].

Nurses play a key role within multidisciplinary teams in helping women with endometriosis manage daily challenges by educating, supporting, and empowering them to adopt self-management strategies [14]. A study performed by Remes, Hakala [15] reported that despite the importance of nursing care in the management of women with endometriosis, high-quality evidence about the effectiveness of organized nursing interventions is still scarce. Most of the previous studies have concentrated on the medical/surgical approach, whereas the role of nursing-led education, counseling, and self-management support interventions has been less commonly investigated. In cases where these interventions have been assessed, results have often been derived from small-scale studies with short-term follow-up or non-randomized designs, and thus, it has been difficult to reach definitive conclusions about their long-term effectiveness.

Nursing interventions may include health education, skill-building, symptom monitoring, and guidance on lifestyle factors such as nutrition, physical activity, and stress management [16]. These interventions have the potential to bridge the gap between clinical treatment and the daily challenges faced by women with endometriosis. However, robust evidence evaluating the effectiveness of nurse-led interventions on self-care and symptom management remains limited [15].

The intervention is theoretically supported by both Orem’s Self-Care Deficit Nursing Theory and the WHO’s Universal Self-Care Framework [17]. Orem’s theory serves as the theoretical basis for the intervention, focusing on the individual’s potential for self-care and the nurse’s role in facilitating self-care when deficits occur. Besides, the WHO’s framework serves to complement this approach by providing a framework for self-care that encompasses a wider public health focus, including health literacy and empowerment. The two frameworks complement each other to provide a sound theoretical base for the intervention. Therefore, this study aims to evaluate the impact of a structured Universal Self-Care Practices module on compliance with self-care and health maintenance behaviors among women with endometriosis. Specifically, it was assessed whether this intervention can lead to a measurable reduction in pain severity.

The researchers hypothesized that:

**H1.**  *Implementing a nursing module based on the WHO Universal Self-Care Framework is expected to improve adherence to health-maintenance behaviors among women with endometriosis at baseline, 1-month, and 3-month follow-ups compared with standard hospital care.*

**H2.**  *Implementing a nursing module based on the WHO Universal Self-Care Framework is expected to significantly reduce pain levels among women with endometriosis at baseline, 1-month, and 3-month follow-ups compared with standard hospital care.*

## 2. Material and Method

### 2.1. Design

A quasi-experimental design was employed in this study.

### 2.2. Settings

The study took place at the Gynecology outpatient clinics of El-Shatby Maternity University Hospital. This site was selected because it is the primary university hospital in Alexandria Governorate, offering obstetrics and gynecological services. Moreover, the high volume of follow-up visits by women made it an appropriate location for the study. Additionally, the attendees at this hospital generally share a similar socioeconomic background, which helped maintain the sample’s homogeneity.

### 2.3. Sample and Sample Size

The study employed a purposive, non-probability sampling technique to recruit a total of 90 women. Eligibility criteria included women of reproductive age with a confirmed diagnosis of endometriosis, who were free from any chronic medical or gynecological comorbidities, and who had not received any pain relief pharmacological interventions. Additionally, all participants provided informed consent and demonstrated willingness to participate in the research.

Sample size was estimated using Epi Info version 7 based on the following parameters: population size of 416 women per month, expected frequency of 50%, 10% acceptable margin of error, and 95% confidence level, yielding a minimum required sample of 78 participants. Although a design effect was not initially incorporated due to the quasi-experimental, single-center nature of the study and absence of cluster sampling, the sample was increased to 90 participants to compensate for potential attrition and enhance statistical power. Participants were equally allocated into a study group (n = 45), which received the nursing intervention, and a control group (n = 45), which received standard hospital management.

### 2.4. Instruments

Three tools were used for data collection, namely Tool (I): Demographic variables, and the women’s history structured interview schedule. This tool was developed and used by the researcher to collect the following data: demographic variables, and women’s history, including age, marital status, occupation, level of education, residence, obstetric and endometriosis history. Tool (II): Universal Self-Care for Endometriosis, which was developed by the researcher based on Orem’s Self-Care Model, in line with the literature [18,19]. It contained questions about Self-Care to meet women’s collective fundamentals. It was composed of 7 items as follows: Personal hygiene (5 items), clothes (2 items), diet (11 items), Fluid intake (3 items), rest and sleep (3 items), exercises (2 items), and drug administration (5 items).

It was employed to assess Self-Care at three time points: before the intervention (pre-test), one month following the intervention (post-test), and three months after the intervention (follow-up). The woman’s response for each item varied between never done 1, sometimes 2, and always 3. The total score of Self-Care ranged between 31 and 93, which ranked as: Good Self-Care: ≥75% (≥70), Fair Self-Care: 50–<75% (46–<70), Poor Self-Care: <50% (<46). Tool III: Visual Analog Scale (VAS), which was originally developed by Katz and Melzack [20]. It was utilized by the researcher to evaluate the severity of pain associated with endometriosis, including dysmenorrhea, dyspareunia, dysuria, dysphasia, and pelvic pain. The VAS is a self-reported measurement tool consisting of a horizontal line designed for patients to subjectively rate their pain intensity. This scale ranges from 0 to 10, where 0 indicates no pain, and 10 corresponds to the most extreme, unbearable pain.

The scale is segmented into intervals, with descriptors such as mild, moderate, and severe pain assigned approximately every 3 cm along the line. Participants were instructed to mark the point on the line that best represented their pain intensity [21]. Pain severity was categorized based on the total score as follows: No pain 0, Mild pain from 1 to 3, Moderate pain from 4 to 6, Severe pain from 7 to 9, and Unbearable pain = 10.

### 2.5. Ethical Considerations

Ethical approval was obtained from the Research Ethics Committee at the Faculty of Nursing, Alexandria University (IRB ref #. IRB00013620). An official request letter was sent from the faculty to the relevant authorities at the study site to secure permission for data collection after explaining the study objectives. Before data collection, and following a comprehensive explanation of the study’s purpose, all participants provided written informed consent, witnessed by the head nurse. Participants were explicitly informed that their participation was entirely voluntary and that they had the right to withdraw from the study at any time without any effect on the medical care they received at the hospital. Confidentiality and privacy were strictly maintained throughout the study, and all collected data were securely handled and protected to ensure anonymity and safeguard participant information.

### 2.6. Pilot Study

A pilot study was conducted among nine women, who were not part of the main study sample, to assess the clarity, relevance, and feasibility of the instruments. The results showed that some minor changes were required to improve understanding and usability. The first instrument, designed by the researcher, was found to be valid by five experts, whereas the second and third instruments, translated into Arabic, were found to be valid by five specialists. The reliability of the second and third instruments was also checked.

### 2.7. Data Collection Procedure

Participants were randomly assigned to two equal groups (n = 45 per group) using a simple randomization procedure. The control group received conventional hospital care for endometriosis, while the study group received a nursing intervention designed using the WHO Universal Self-Care Response. To prevent contamination, data from the control group were gathered before starting the intervention in the study group. Both groups were assessed at three points in time: baseline, one month, and three months. Outcome measurement was done using standardized instruments (II and III); nevertheless, owing to the type of intervention, it was impossible to blind the participants. The self-care instrument designed by the researchers was content validated by five experts, and internal consistency was established using Cronbach’s alpha coefficient (α = 0.75).

The study employed three established instruments to measure the outcomes, in addition to the self-care tool designed by the researcher (validated for content by five experts and for internal consistency). The data were gathered at three points in time: baseline, one month post-intervention, and three months post-intervention. While the data was gathered through repeated measures, the pre-test and post-test comparisons were emphasized to enable a comparison between the intervention and control groups at the three points in time. Repeated-measures analysis of variance (ANOVA) was considered, but due to the quasi-experimental nature of the study, the unequal variance assumptions, and the emphasis on the between-group comparisons at the three points in time, separate comparisons at each point in time using paired and independent tests were conducted. Post hoc power analysis and effect size calculations were conducted for all outcome measures to ensure sufficient statistical power and to estimate the size of the observed effects.

### 2.8. Components of the Self-Care Module

**Figure 1:**
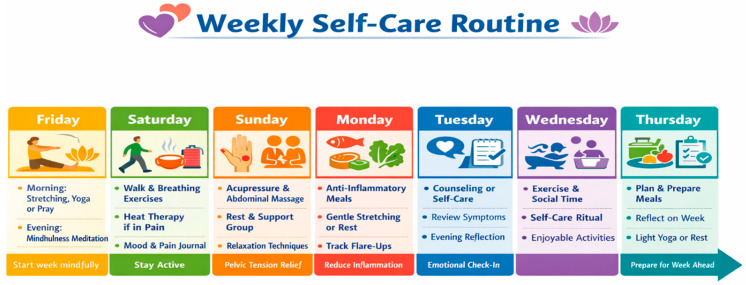
Weekly self-care routine Schedule.

The 12-week module consisted of seven integrated components, which were gradually modified according to participant feedback and symptom progression. These components included health education on endometriosis, symptom recognition, and trigger factors to improve self-efficacy; daily symptom and pain assessment using standardized symptom and pain journals; lifestyle and behavioral interventions for anti-inflammatory diets, hydration, dietary supplements, low-intensity exercise, sleep hygiene, and stress management strategies like mindfulness, breathing exercises, progressive muscle relaxation, acupressure, and heat therapy. Participants were educated on the safe use of physical and assistive therapies, provided psychosocial support through facilitated peer interaction, journaling, and counseling, and utilized digital health platforms for symptom management and telemedicine sessions. Lastly, the program was integrated with medical support, with proper referral mechanisms for severe or deteriorating symptoms (Figure 1).

### 2.9. Program Delivery and Support

The program was delivered in a hybrid format combining self-guided materials, weekly group check-ins (virtual or physical), and optional individualized coaching. Materials were adapted to the local context, ensuring cultural relevance and accessibility. A sample weekly routine was provided to encourage consistent implementation. The intervention was designed in accordance with the WHO conceptual framework on self-care (WHO, 2023) [3], complemented by clinical guidelines from NICE (NG73), ISGE, and patient-informed practices documented in peer-reviewed literature. Emphasis was placed on the biopsychosocial model of pain and the chronic illness self-management framework. This weekly routine was provided to all participants enrolled in the intervention group. It was designed to support consistent engagement with self-care activities across physical, emotional, and lifestyle domains. Participants were encouraged to adapt the schedule based on their menstrual cycle, symptom severity, and daily responsibilities.

### 2.10. Data Analysis

Once the data collection process was finished, all data were coded and entered into SPSS version 20 for analysis. Descriptive statistics were used to describe the participants, while inferential statistics were used to determine the impact of the module on compliance and pain relief. Normality tests were conducted on continuous variables using the Shapiro–Wilk test. Based on the results, t-tests or Wilcoxon tests were used for normally distributed data, while Mann–Whitney tests were used for non-normal data. The level of significance was set at *p* ≤ 0.05, and comparisons were made between the intervention and control groups before and after the intervention.

## 3. Results

The mean age of the participants was comparable between the study group (33.11 ± 5.08 years) and the control group (32.44 ± 4.75 years), with no significant difference (*p* = 0.651). The educational attainment of the participants was also similar, with almost equal proportions of the study group having completed secondary education compared to the control group, and 22.2% of participants in both groups having completed university-level or higher education (χ^2^ = 3.721, *p* = 0.293). In relation to occupation, 60% of the study group and 44.4% of the control group were workers, with the remaining participants being housewives. The proportion of married and single participants was also equal in both groups, with about half of the participants being single and the other half being married in both groups (χ^2^ = 6.898, *p* = 0.075). Most participants came from urban areas (77.8% vs. 71.1%), with no significant difference (χ^2^ = 0.526, *p* = 0.468). In general, there were no statistically significant differences in the socio-demographic characteristics of the participants, which were homogeneous in both groups (Table 1).

The duration of endometriosis in Table 2 was not significantly different between the groups, with the majority of the study group having the condition for less than 3 years (42.2%), while almost half of the control group had it for more than 7 years (46.7%), although not statistically significant (χ^2^ = 3.080, *p* = 0.214). The time of diagnosis was also similar, with almost half of both groups diagnosed between 2 and 3 years, and no statistically significant differences (χ^2^ = 1.081, *p* = 0.582). The distribution of disease stages and symptoms such as dysmenorrhea, back and abdominal pain (100%), fatigue, dyspareunia, pelvic pain, and gastrointestinal symptoms was also similar between the groups, with no statistically significant differences. The equality of the groups at baseline was thus established.

With respect to universal self-care practices, the study group demonstrated significant improvement over time compared to the control group. By the third month, the percentage of adherence to bathing, hand washing, and perineal hygiene practices was 100%, 91.1%, and 91.1%, respectively, compared to the control group. The use of cotton underwear and loose-fitting clothing, a healthy diet, daily fruit and vegetable consumption, fatty fish, nuts, and plant oil consumption, and dietary restrictions (reduction in red meat, fat, sugar, salt, and carbohydrates) increased significantly in the study group compared to the control group. Hydration, soft drink and caffeine avoidance, adequate sleep, regular daily activities, light exercise, medication compliance, and supplement use (omega-3, vitamin D, and multivitamins) also increased significantly. In general, the study group showed significant enhancement of nearly all self-care practices by the third month compared to the control group, which showed little change (Table 3).

Correlation analysis revealed a strong, significant negative correlation between age and total self-care practice at the end of the intervention (r = −0.633, *p* < 0.001), indicating that younger individuals had better self-care practices. However, the strength of this correlation gradually reduced and became non-significant at one month (r = −0.079, *p* = 0.457) and three months (r = 0.083, *p* = 0.439), indicating that the effect of age gradually reduced. As shown in Table 4, there was no significant difference in the mean self-care scores between the study (M = 55.5 ± 5.99) and control (M = 55.2 ± 6.28) groups at the end of the intervention (*t* = −0.206, *p* = 0.310). However, the study group showed significant improvement at one month (M = 69.2 ± 11.6 vs. 58.3 ± 8.83; *t* = −4.93, *p* = 0.001, 95% CI −15.1 to −6.42) and three months (M = 76.4 ± 16.5 vs. 61.5 ± 12.2; *t* = −4.89, *p* = 0.001, 95% CI −21.1 to −8.89) after the intervention. The intervention resulted in progressive improvements in universal self-care practices.

Correlation analysis showed significant relationships between scores of self-care practice and VAS symptom scores. There were strong and positive correlations between self-care practice scores at one- and three-month post-intervention (r = 0.96, *p* < 0.001), showing progressive improvement. There were also significant negative correlations between self-care practice and VAS symptom scores, especially at one- and three-month post-intervention (r = −0.70 and r = −0.83, *p* < 0.001), showing that higher levels of self-care practice were associated with lower levels of symptoms. VAS symptom scores were also positively correlated (e.g., VAS1-VAS2: r = 0.52; VAS2-VAS3: r = 0.86, *p* < 0.001), showing stable symptom patterns. These results suggest a relationship between improved self-care practices and decreased symptom severity, indicating the effectiveness of the intervention (Table 5).

## 4. Discussion

The findings of the study show that there is a significant improvement in the compliance of self-care and pain management in the intervention group due to the nursing intervention, and this is observed at both one-month and three-month follow-ups.

The findings performed by Alasser, Eshra [22] supported the current study results, as it was mentioned that the study group experienced a highly statistically significant reduction in the intensity of pain linked to endometriosis. Moreover, a significant statistical difference was observed in the self-care practices score of women for reducing symptoms associated with endometriosis following the nursing guidelines as opposed to their pre-nursing guidance.

Likewise, this aligns with the findings of ElSewefy, Khairy [23], who reported a notable improvement in women’s knowledge and self-care practices following participation in an educational program. The study emphasized the vital role of healthcare providers in supporting patients’ management of endometriosis, helping them cope with the condition, and developing effective strategies for symptom control.

The findings of the current study align with the existing systematic review that was performed by Tennfjord and Gabrielsen [24], which highlights the potential benefits of physical activity and exercise as non-pharmacological strategies for managing endometriosis-related symptoms. A recent systematic review summarized evidence from four interventional studies involving 129 women, though one study was excluded due to methodological flaws, resulting in a final sample of 109 participants. Notably, each of the included studies reported some degree of improvement in key symptom domains, such as pain intensity, stress levels, psychological well-being, and self-image. These outcomes suggest that incorporating structured physical activity into self-care programs may contribute positively to both physical and emotional aspects of endometriosis management [25,26,27].

Recent findings indicate that dietary changes can help alleviate endometriosis symptoms by fighting inflammation and regulating estrogen levels. Anti-inflammatory diets that include plenty of fruits, vegetables, whole grains, and omega-3 fatty acids, as well as cutting back on red meat and processed foods, are increasingly being seen as a complementary therapy in self-care practices.

The statistically significant reduction in reported pain levels in the study group at both one- and three-month post-intervention indicates that the nursing program adopted not only increased self-care but also translated into meaningful clinical outcomes. This is consistent with prior findings suggesting that multidimensional self-care can alleviate pain severity in chronic conditions. This can be understood by addressing biopsychosocial factors [28]. The fact that the pain was reduced for three months shows that the program led to long-term behavioral changes. On the other hand, the control group, which received routine care, did not show any significant improvements. This highlights the importance of individualized nursing interventions that are evidence-based and tailored to the needs of each woman.

The observed improvement in self-care compliance may be explained by the program’s incorporation of women’s learning principles, patient-centered education, and follow-up reinforcement sessions. These elements align with the Health Belief Model, which suggests that enhanced perceived benefits, greater self-efficacy, and cues to action, such as follow-up by nurses, can significantly promote adherence to healthy behaviors (Alharbi & Shehzad, 2021) [29]. Besides, the observed positive outcomes can be attributed to a number of mechanisms. The intervention would have certainly increased self-efficacy by providing the women with skills and strategies to cope with their symptoms [30]. In the same line, a study performed by Kvåle, Tokovska [31] reported that improved health literacy would have empowered women to understand their condition and make appropriate lifestyle choices. The empowerment approach would have also encouraged autonomy and self-care practices, leading to sustained improvements in pain and compliance outcomes.

Importantly, these findings support the incorporation of WHO-endorsed self-care strategies within nursing practice as a cost-effective and scalable means of improving outcomes for women with endometriosis. This aligns with global health priorities advocating for greater integration of self-care into primary health care systems (WHO, 2021) [32].

### 4.1. Strengths and Limitations

This study’s strengths include the integration of the WHO’s Universal Self-Care Framework into a structured, nursing-led module, the use of a quasi-experimental design for reliable comparison, and follow-up assessments confirming sustained improvements. Additionally, qualitative insights could provide a deeper understanding of patient experiences and perceived barriers to self-care adherence. These elements demonstrate the effectiveness of evidence-based, nurse-led interventions in enhancing self-care and reducing pain among women with endometriosis. While the results are promising, several limitations should be acknowledged. The follow-up period was limited to three months; thus, long-term sustainability of compliance and pain reduction remains uncertain. Future studies should explore longer follow-up durations and assess potential mediating factors such as psychological well-being and quality of life. The use of purposeful, non-probability sampling may limit generalizability. Future studies should use larger, randomized samples to enhance external validity. However, the quasi-experimental design does require careful attention. Although the group equivalence was established at the beginning of the study, the lack of full randomization and blinding may pose a potential threat to selection bias. Although the results do offer preliminary support for the effectiveness of the intervention, future studies with full randomization and longer follow-up periods are recommended. Additionally, the possibility of social desirability bias should also be taken into consideration, as practices of self-care and symptoms were based on self-report data, which could be prone to the influence of the participants’ need to provide positive responses. Moreover, the application of a locally developed self-care instrument, although validated and reliable, could pose limitations in terms of comparability with other studies and the generalizability of the results.

### 4.2. Implications and Recommendations

Future research should examine the role of particular psychosocial variables, like stress, coping, social support, and health literacy, which might play a part in self-care and symptom management in women with endometriosis. Research methods may include longitudinal studies, mixed-method studies, or randomized controlled trials to assess the effectiveness of interventions over time. It is suggested that personalized, nursing-based self-care programs, developed to meet the specific needs of each woman, be incorporated into the management of endometriosis, with a focus on continuous education and reinforcement of self-care behaviors. From a nursing practice perspective, the implications of this research highlight the importance of the nurse’s role in self-management and empowering women. In a broader sense, the adoption of the WHO Universal Self-Care Framework in the policies and guidelines of gynecological care may improve the quality, consistency, and effectiveness of care. Additionally, future studies should include longer follow-up periods of 6–12 months to better assess the persistence of behavior changes.

## 5. Conclusions

In conclusion, this study reinforces the critical role of nursing in implementing self-care interventions grounded in WHO guidelines to support women with endometriosis. The significant improvements in compliance and pain reduction observed in the study group affirm the value of nursing-led programs. Integrating such interventions into routine gynecological care may enhance self-management capacity and improve the overall quality of life for affected women. The study hypotheses were accepted.

## Figures and Tables

**Table 1:** Distribution of the study groups according to their socio-demographic characteristics (n = 90).

Socio-Demographic Characteristics	Study Group		Control Group		χ^2^	*p*
	No	%	No	%		
Age	33.11 ± 5.082			32.44 ± 4.746	0.338	0.651
25–35	37	82.2	39	86.7		
˃35–45	8	17.8	6	13.3		
Education level					3.721	0.293
Illiterate	5	11.1	9	20.0		
Primary	8	17.8	12	26.7		
Secondary	22	48.9	14	31.1		
University or higher	10	22.2	10	22.2		
Occupation					2.954	0.228
Housewife	18	40.0	25	55.6		
Worker	27	60.0	20	44.4		
Marital status					6.898	0.075
Single	22	48.9	20	44.4		
Married	23	51.1	25	55.6		
Current residence					0.526	0.468
Urban	35	77.8	32	71.1		
Rural	10	22.2	13	28.9		

**Table 2:** Distribution of the study groups according to their endometriosis history (n = 90).

Endometriosis History	Study Group		Control Group		X^2^	*p*
	No	%	No	%		
Duration of endometriosis (years)					3.080	0.214
<3 years	19	42.2	15	33.3		
4–6 years	13	28.9	9	20.0		
>7 years	13	28.9	21	46.7		
Diagnosis timing					1.081	0.582
1 year	14	31.1	10	22.2		
2–3 years	21	46.7	22	48.9		
>3 years	10	22.2	13	28.9		
Endometriosis stage					2.198	0.333
Mild	14	31.1	10	22.2		
Moderate	13	28.9	10	22.2		
Sever	18	40.0	25	55.6		
Symptoms associated with endometriosis						
Dysmenorrhea					1.52	0.423
Yes	45	100	45	100		
No	0	0	0	0		
Fatigue					0.909	0.340
Yes	31	68.9	35	77.8		
No	14	31.1	10	22.2		
Dyspareunia					1.353	0.245
Yes	36	80	40	88.9		
No	9	20	5	11.1		
Back pain					1.243	0.321
Yes	45	100	45	100		
No	0	0	0	0		
Pelvic pain					0.051	0.822
Yes	31	68.9	30	66.7		
No	14	31.1	15	33.3		
Abdominal pain					1.523	0.253
Yes	45	100	45	100		
No	0	0	0	0		
Digestive symptoms					0.051	0.822
Yes	31	68.9	30	66.7		
No	14	31.1	15	33.3		

Note: *p* is statistically significant at ≤0.05.

**Table 3:** Distribution of the study and control groups according to their universal self-care practices regarding endometriosis (n = 90).

Universal Self-Care Practices	Immediately				After 1 Month				After 3 Months			
	Study		Control		Study		Control		Study		Control	
	No.	%	No.	%	No.	%	No.	%	No.	%	No.	%
Daily bath												
Never	0	0	0	0	0	0	0	0	0	0	0	0
Sometimes	10	22.2	13	28.9	5	11.1	13	28.9	0	0	4	8.9
Always	35	77.8	32	71.1	40	88.9	32	71.1	45	100	41	91.1
Hand washing												
Never	0	0	0	0	0	0	0	0	0	0	0	0
Sometimes	18	40.0	25	55.6	9	20.0	21	46.7	4	8.9	12	26.7
Always	27	60.0	20	44.4	36	80.0	24	53.3	41	91.1	33	73.3
Shower												
Never	0	0	0	0	0	0	0	0	0	0	0	0
Sometimes	23	51.1	25	55.6	9	20.0	21	46.7	4	8.9	12	26.7
Always	22	48.9	20	44.4	36	80.0	24	53.3	41	91.1	33	73.3
Perineum care												
Never	0	0	0	0	0	0	0	0	0	0	0	0
Sometimes	23	51.1	30	66.7	9	20.0	26	57.8	4	8.9	17	37.8
Always	22	48.9	15	33.3	36	80.0	19	42.2	41	91.1	28	62.2
Cotton underwear												
Never	0	0	0	0	0	0	0	0	0	0	0	0
Sometimes	23	51.1	15	33.3	9	20.0	26	57.8	4	8.9	17	37.8
Always	22	48.9	36	9.0	36	80.0	19	42.2	41	91.1	28	62.2
Wide clothes												
Never	0	0	5	11.1	0	0	5	11.1	0	0	5	11.1
Sometimes	31	68.9	31	68.9	23	51.1	27	60.0	22	48.9	32	71.1
Always	14	31.1	9	20.0	22	48.9	13	28.9	23	51.1	8	17.8
Avoid high heels												
Never	13	28.9	12	26.7	8	17.8	12	26.7	8	17.8	12	26.7
Sometimes	18	40.0	19	42.2	28	62.2	24	53.3	18	40.0	24	53.3
Always	14	31.1	14	31.1	9	20.0	9	20.0	19	42.2	9	20.0
Balanced diet												
Never	9	20.0	8	17.8	0	0	0	0	0	0	0	0
Sometimes	31	68.9	33	73.3	27	60.0	37	82.2	22	48.9	37	82.2
Always	5	11.1	4	8.9	18	40.0	8	17.8	23	51.1	8	17.8
Fruits and vegetables												
Never	4	4	8	17.8	13	28.9	22	48.9	0	0	0	0
Sometimes	41	41	37	82.2	23	51.1	23	51.1	17	37.8	32	71.1
Always	0	0	0	0	9	20.0	0	0	28	62.2	13	28.9
Fatty fish												
Never	21	46.7	21	46.7	13	28.9	22	48.9	8	17.8	17	37.8
Sometimes	24	53.3	24	53.3	23	51.1	23	51.1	14	31.1	24	53.3
Always	0	0	0	0	9	20.0	0	0	23	51.1	4	8.9
Nuts												
Never	22	22	21	46.7	13	28.9	22	48.9	8	17.8	22	48.9
Sometimes	23	23	24	53.3	23	51.1	23	51.1	14	31.1	19	42.2
Always	0	0	0	0	9	20.0	0	0	23	51.1	4	8.9
Plant oil												
Never	21	46.7	22	48.9	13	28.9	18	40.0	8	17.8	18	40.0
Sometimes	24	53.3	23	51.1	23	51.1	27	60.0	14	31.1	23	51.1
Always	0	0	0	0	9	20.0	0	0	23	51.1	4	8.9
Reduce red meat												
Never	26	57.8	26	57.8	8	17.8	17	37.8	8	17.8	22	48.9
Sometimes	19	42.2	19	42.2	32	71.1	28	62.2	14	31.1	19	42.2
Always	0	0	0	0	5	11.1	0	0	23	51.1	4	8.9
Reduction in animal products												
Never	27	60.0	27	60.0	4	8.9	19	42.2	4	8.9	19	42.2
Sometimes	18	40.0	18	40.0	37	82.2	22	48.9	22	48.9	22	48.9
Always	0	0	0	0	4	8.9	4	8.9	19	42.2	4	8.9
Fat reduction												
Never	21	46.7	21	46.7	8	17.8	17	37.8	8	17.8	17	37.8
Sometimes	24	53.3	24	53.3	32	71.1	28	62.2	27	60.0	28	62.2
Always	0	0	0	0	5	11.1	0	0	10	22.2	0	0
Salt reduction												
Never	26	57.8	31	68.9	8	17.8	27	60.0	8	17.8	27	60.0
Sometimes	19	42.2	14	31.1	32	71.1	18	40.0	14	31.1	14	31.1
Always	0	0	0	0	5	11.1	0	0	23	51.1	4	8.9
Sugar reduction												
Never	31	68.9	31	68.9	8	17.8	27	60.0	8	17.8	27	60.0
Sometimes	14	31.1	14	31.1	37	82.2	18	40.0	14	31.1	14	31.1
Always	0	0	0	0	0	0	0	0	23	51.1	4	8.9
Carbohydrate reduction												
Never	21	46.7	21	46.7	8	17.8	17	37.8	8	17.8	17	37.8
Sometimes	24	53.3	24	53.3	37	82.2	28	62.2	14	31.1	24	53.3
Always	0	0	0	0	0	0	0	0	23	51.1	4	8.9
Enough water												
Never	4	8.9	8	17.8	0	0	0	0	0	0	0	0
Sometimes	41	91.1	37	82.2	36	80.0	41	91.1	22	48.9	41	91.1
Always	0	0	0	0	9	20.0	4	8.9	23	51.1	4	8.9
Soft drinks												
Never	26	57.8	26	57.8	13	28.9	22	48.9	8	17.8	22	48.9
Sometimes	14	31.1	14	31.1	28	62.2	18	40.0	14	31.1	14	31.1
Always	5	11.1	5	11.1	4	8.9	5	11.1	23	51.1	9	20.0
Caffeine avoidance												
Never	31	68.9	31	68.9	13	28.9	27	60.0	8	17.8	27	60.0
Sometimes	14	31.1	14	31.1	28	62.2	18	40.0	14	31.1	14	31.1
Always	0	0	0	0	4	8.9	0	0	23	51.1	4	8.9
Night sleep												
Never	0	0	0	0	0	0	0	0	0	0	0	0
Sometimes	45	100	45	100	36	80.0	45	100	22	48.9	41	91.1
Always	0	0	0	0	9	20.0	0	0	23	51.1	4	8.9
Naps												
Never	0	0	0	0	0	0	0	0	0	0	0	0
Sometimes	37	82.2	40	88.9	32	71.1	40	88.9	18	40.0	36	80.0
Always	8	17.8	5	11.1	13	28.9	5	11.1	27	60.0	9	20.0
Daily routine												
Never	0	0	0	0	0	0	0	0	0	0	0	0
Sometimes	37	82.2	40	88.9	28	62.2	36	80.0	18	40.0	36	80.0
Always	8	17.8	5	11.1	17	37.8	9	20.0	27	60.0	9	20.0
Daily light exercises												
Never	5	11.1	4	8.9	0	0	4	8.9	0	0	4	8.9
Sometimes	32	71.1	36	80.0	23	51.1	32	71.1	18	40.0	32	71.1
Always	8	17.8	5	11.1	22	48.9	9	20.0	27	60.0	9	20.0
Heavy exercises												
Never	31	68.9	31	68.9	9	20.0	19	42.2	4	8.9	19	42.2
Sometimes	14	31.1	14	31.1	36	80.0	26	57.8	26	57.8	17	37.8
Always	0	0	0	0	0	0	0	0	15	33.3	9	20.0
Prescribed medication												
Never	0	0	0	0	0	0	0	0	0	0	0	0
Sometimes	40	88.9	40	88.9	27	60.0	36	80.0	22	48.9	36	80.0
Always	5	11.1	5	11.1	18	40.0	9	20.0	23	51.1	9	20.0
Avoid unprescribed medication												
Never	30	66.7	26	57.8	17	37.8	22	48.9	1	2.2	21	46.7
Sometimes	10	22.2	14	31.1	23	51.1	18	40.0	16	35.6	6	13.3
Always	5	11.1	5	11.1	5	11.1	5	11.1	28	62.2	18	40.0
Omega-3 consumption												
Never	35	77.8	31	68.9	17	37.8	27	60	17	37.8	27	60.0
Sometimes	10	22.2	14	31.1	23	51.1	18	40	5	11.1	14	31.1
Always	0	0	0	0	5	11.1	0	0	23	51.1	4	8.9
Vitamin D consumption												
Never	40	88.9	36	80.0	17	37.8	32	71.1	17	37.8	32	71.1
Sometimes	5	11.1	9	20.0	23	51.1	13	28.9	5	11.1	9	20.0
Always	0	0	0	0	5	11.1	0	0	23	51.1	4	8.9
Multivitamins consumption												
Never	22	48.9	21	46.7	13	28.9	17	37.8	13	28.9	17	37.8
Sometimes	23	51.1	24	53.3	23	51.1	28	62.2	9	20.0	24	53.3
Always	0	0	0	0	9	20.0	0	0	23	51.1	4	8.9

**Table 4:** Differences in Universal Self-Care Practices Between Study and Control Groups at Immediate, One-Month, and Three-Month Intervals.

	Groups	M ± SD	*t*-Test	*p*-Value	95% Confidence Interval of the Difference
The total score of universal self-care practices immediately	Study group	55.5 ± 5.99	−0.206	0.310	−2.83–2.305
	Control group	55.2 ± 6.28			
Total score of universal self-care practices immediately after 1 month	Study group	69.2 ± 11.6	−4.93	0.001 *	−15.1–−6.42
	Control group	58.3 ± 8.83			
Total score of universal self-care practices immediately after 3 months	Study group	76.4 ± 16.5	−4.89	0.001 *	−21.1–−8.89
	Control group	61.5 ± 12.2			

Note: * *p* is statistically significant at ≤0.05.

**Table 5:** Pearson Correlations Between Self-Care Practice Scores and VAS Scores Over Time.

No.	Variables	1	2	3	4	5	6
1	Self-care (Immediate)	—	0.03	−0.01	−0.18	−0.33 **	−0.27 **
2	Self-care (1 month)		—	0.96 **	−0.19	−0.70 **	−0.88 **
3	Self-care (3 months)			—	−0.10	−0.65 **	−0.83 **
4	VAS (Immediate)				—	0.52 **	0.20
5	VAS (1 month)					—	0.86 **
6	VAS (3 months)						—

Notes: VAS = Visual Analog Scale, ** *p* < 0.01 (2-tailed).

## Data Availability

The dataset is available on request from the authors, and it is not publicly available due to privacy or ethical restrictions.
